# Augmented Expansion of Treg Cells From Healthy and Autoimmune Subjects *via* Adult Progenitor Cell Co-Culture

**DOI:** 10.3389/fimmu.2021.716606

**Published:** 2021-09-01

**Authors:** James L. Reading, Valerie D. Roobrouck, Caroline M. Hull, Pablo Daniel Becker, Jelle Beyens, Alice Valentin-Torres, Dominic Boardman, Estefania Nova Lamperti, Samantha Stubblefield, Giovanna Lombardi, Robert Deans, Anthony E. Ting, Timothy Tree

**Affiliations:** ^1^Cancer Immunology Unit, Research Department of Haematology, University College London Cancer Institute, London, United Kingdom; ^2^Peter Gorer Department of Immunobiology, School of Immunology and Microbial Sciences, King’s College London, London, United Kingdom; ^3^King’s College London Department of Immunoregulation and Immune Intervention, Guy’s Hospital, London, United Kingdom; ^4^Department of R&D, ReGenesys BV, Leuven, Belgium; ^5^Department of R&D, Athersys Inc., Cleveland, OH, United States; ^6^Department of Surgery, The University of British Columbia, Vancouver, BC, Canada; ^7^ Department of Surgery, BC Children’s Hospital Research Institute, Vancouver, BC, Canada; ^8^Molecular and Translational Immunology Laboratory, Department of Clinical Biochemistry and Immunology, Faculty of Pharmacy, Universidad de Concepcion, Concepcion, Chile; ^9^MRC Centre for Transplantation, Peter Gorer Department of Immunobiology, Faculty of Life Sciences & Medicine, King’s College London, London, United Kingdom; ^10^NIHR Biomedical Research Centre Guys and St Thomas’ NHS Foundation Trust and Kings College London, London, United Kingdom

**Keywords:** *ex vivo* expansion, autoimmune diseases, adult progenitor cells, co-culture, regulatory T (Treg) cells

## Abstract

Recent clinical experience has demonstrated that adoptive regulatory T (Treg) cell therapy is a safe and feasible strategy to suppress immunopathology *via* induction of host tolerance to allo- and autoantigens. However, clinical trials continue to be compromised due to an inability to manufacture a sufficient Treg cell dose. Multipotent adult progenitor cells (MAPC^Ⓡ^) promote Treg cell differentiation *in vitro*, suggesting they may be repurposed to enhance *ex vivo* expansion of Tregs for adoptive cellular therapy. Here, we use a Good Manufacturing Practice (GMP) compatible Treg expansion platform to demonstrate that MAPC cell-co-cultured Tregs (MulTreg) exhibit a log-fold increase in yield across two independent cohorts, reducing time to target dose by an average of 30%. Enhanced expansion is coupled to a distinct Treg cell-intrinsic transcriptional program characterized by elevated expression of replication-related genes (*CDK1, PLK1, CDC20*), downregulation of progenitor and lymph node-homing molecules (*LEF1 CCR7, SELL*) and induction of intestinal and inflammatory tissue migratory markers (*ITGA4, CXCR1*) consistent with expression of a gut homing (CCR7lo β_7_hi) phenotype. Importantly, we find that MulTreg are more readily expanded from patients with autoimmune disease compared to matched Treg lines, suggesting clinical utility in gut and/or T helper type1 (Th1)-driven pathology associated with autoimmunity or transplantation. Relative to expanded Tregs, MulTreg retain equivalent and robust purity, FoxP3 Treg-Specific Demethylated Region (TSDR) demethylation, nominal effector cytokine production and potent suppression of Th1-driven antigen specific and polyclonal responses *in vitro* and xeno Graft vs Host Disease (xGvHD) *in vivo*. These data support the use of MAPC cell co-culture in adoptive Treg therapy platforms as a means to rescue expansion failure and reduce the time required to manufacture a stable, potently suppressive product.

## Introduction

Regulatory T cells (Tregs) are pivotal regulators of immune responses, maintaining self-tolerance, homeostasis, and controlling excessive immune activation through a spectrum of cell-mediated and soluble mechanisms. The best characterized subset of Tregs are those defined by constitutive expression of CD25 and FOXP3, the master regulator of their suppressive phenotype and function (1). Tregs play a key role in the prevention of autoimmune diseases, allergies, infection-induced organ pathology, transplant rejection and GvHD. Based on encouraging results in pre-clinical models, adoptive transfer of *ex vivo* expanded Tregs is seen as a promising therapeutic strategy to restore immune balance and promote tolerance in individuals undergoing hematopoietic stem cell- and solid organ transplantation or suffering from autoimmune diseases such as Crohn’s disease (CD) and type 1 diabetes (T1D) (2). Recently, early phase clinical trials have demonstrated that adoptive Treg cell therapy is safe and feasible (3). However, many clinical trials have been compromised due to manufacturing challenges, primarily in the isolation of pure Tregs and *ex vivo* expansion to produce sufficient cell yields for a clinical dose (3–5).

We recently developed a GMP-compatible platform at King’s College London (KCL) for the isolation and expansion of Tregs for adoptive cell therapy that has been trialed as a method to promote renal allograft tolerance as part of the ONEstudy (6, 7) and the ThRIL study (8, 9). A key feature of this platform is the inclusion of the mTOR inhibitor Rapamycin, which prevents effector T cell outgrowth (10–12). In parallel, we have explored the immunomodulatory potential of MAPC cells, an adult, adherent bone-marrow derived stromal cell that is under clinical investigation for numerous indications (13–16) and determined that these cells suppress effector T cell function and promote Treg induction in murine and human models of autoimmunity, transplantation and injury (17–20). We hypothesized that the immunomodulatory potential of these two clinical grade cell therapies may synergize such that Treg abundance or function could be enhanced in the presence of MAPC cells; thereby establishing superior protocols to advance Treg manufacture for adoptive cell therapy.

## Methods

### MAPC Cells

MAPC cells used throughout the majority of this study were manufactured by Athersys (Cleveland, OH) using femoral bone marrow aspirates from fully consented donors and processed according to previously described methods (21). The cells were subjected to several quality control tests to guarantee the quality of the expanded cell product (post-thaw viability, flow cytometric analysis of positive/negative surface markers, cytokine secretion for their angiogenic capacity and a T cell proliferation assay to evaluate their immunosuppressive function). MAPC cells were isolated from the bone marrow of a healthy volunteer after obtaining informed consent in accordance with the guidelines of an Institutional Review Board.

### Primary Cell Culture

**Figure 1:**
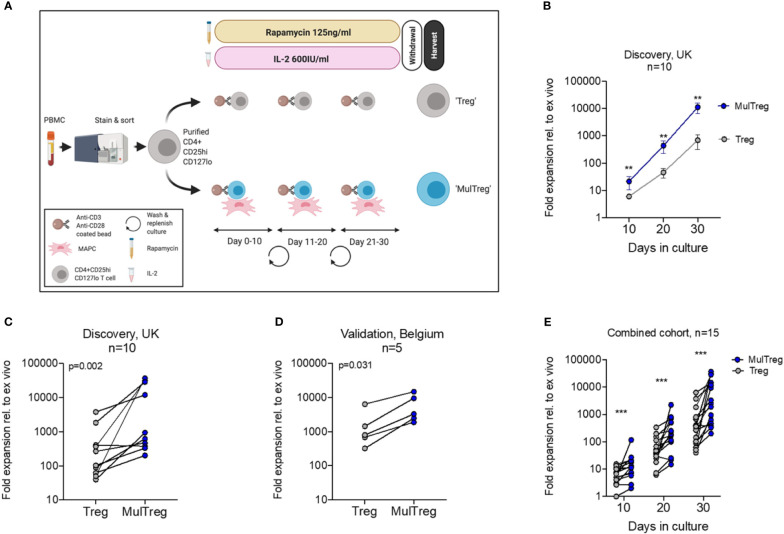
Treg and MulTreg isolation and expansion. **(A)** Schematic of the expansion protocol for Treg and MulTreg. **(B)** The fold expansion of Treg and MulTreg lines vs *ex vivo* (fresh Tregs plated post sort) was calculated for each donor at each time point. Datapoints represent the mean +/- SEM for 10 donors. **(C)** Fold expansion (from *ex vivo*) at d30 for the UK (KCL) cohort (Treg and MulTreg lines grown from 10 individuals). **(D)** Average fold expansion (from *ex vivo*) at d30 for the validation (Belgian) cohort (Treg and MulTreg lines grown from 5 individuals). **(E)** Fold expansion in MulTreg versus Treg expansion was calculated for each donor at each time point in the combined cohort: Treg and MulTreg lines grown from 15 individuals. All p and q values from single or multiple (corrected) matched-pairs Wilcoxon-signed rank test. **q < 0.01, ***q < 0.0005.

PBMC were isolated from fresh blood of consented healthy volunteers by density gradient centrifugation (Lymphoprep, Axis Shield, Oslo, Norway). Stored PBMC samples from individuals with recent onset T1D and HD were included in this study and was approved by the UK National Research Ethics Service (REC# 08/H0805/14). Written informed consent was obtained from all participants prior to inclusion in the study. Frozen PBMCs from Crohn’s disease patients were obtained from Precision for Medicine or Stem Cell Technologies (**Table S1**). Effector (hereafter Teff, CD25^lo^) and regulatory (CD127^lo^ CD25^hi^) T cells were 2-way sorted from PBMC pre-gated on CD4^+^CD14^-^ viable lymphocytes using a FACS Aria (BD) and the antibodies listed in **Supplementary Material** procured from Biolegend or BD. To establish Treg lines, CD127^lo^ CD25^hi^ CD4^+^ lymphocytes were plated at 5x10^4^/well of round bottom 96-well plates (Corning) in 200μl 0.2μM filtered (Sartorius, Terumo) complete media consisting of X-VIVO 15 media (Lonza) containing 100μg/ml Penicillin-streptomycin, 100μg/ml amphotericin B (both from Sigma Aldrich), 125ng/ml Rapamycin (Rapamune, Pfizer), 600 IU/ml IL-2 (Proleukin, Chiron), and 5% heat inactivated human AB serum (Sigma Aldrich) and incubated at 37°C, 5% CO_2_. Cells were stimulated with CD3/28 beads (2:1 bead to cell ratio) (Dynabeads, Life Technologies) for 72h then harvested, pooled, and transferred to flat bottom 96 well or 48 well plates at a density of 1x10^6^/ml. Cells were maintained by feeding with complete media 2-3 times during days 3-10 and transferred to T75 or T125 when cell numbers exceeded 3x10^7^. This 10-day expansion was repeated twice for a total of 30 days in culture. After 30 days residual beads were removed *via* magnet (Dynal, Invitrogen), cells were then washed once in X-VIVO 15 media and replated at 2x10^6^/ml in X-VIVO 15 media containing 2.5% heat inactivated human AB serum for 48h (withdrawal). Autologous MulTreg lines were generated in parallel from the same suspension of sorted Tregs under identical conditions with the exception of adding 1:10 MAPC : Treg on day 0 of each round immediately prior to CD3/28 stimulation. To prepare MAPC cells, the cells were thawed and washed once (500 x g/5 mins), then resuspended in complete Treg media. MAPC cells adhered to flat bottom plates and flasks and were absent from final preparations of MulTreg lines as confirmed by microscopy and flow cytometry using anti-CD105 staining on ungated events (data not shown). Following 30 days plus 48h withdrawal, yields were determined by the average of five counts from a single cell suspension and fresh Treg/MulTreg lines were analyzed by flow cytometry or Sorted Teff cells were cryopreserved without expansion at day 0 cryopreserved. Graphic in **Figure 1A** generated using BioRender.com.

### Flow Cytometry

Dead cells were excluded with Fixable Live/dead blue (UV450 Molecular probes, Invitrogen, Life technologies) or 7AAD (BD). Bespoke overlapping Treg panels were constructed using the fluorochrome-labeled antibodies listed in the **Supplementary Methods** (BD, Biolegend). Intracellular staining was performed using the FoxP3 staining kit (eBiosciences) according to the manufacturers’ instructions. Flow cytometry acquisition was conducted using a BD LSR Fortessa (BD) or BD Celesta (BD), cell sorting completed using the BD FACS ARIA, all equipped with FACS Diva software (v6.0- 8.0 (BD Biosciences). Data was analyzed using Flowjo X (Treestar, Ashland, OR).

### Data Analysis

Data was analyzed using Prism v8-9 software (Graphpad), and after checking normal distribution of the data, the appropriate statistical test was used for parametric or non-parametric calculations (indicated in the figure legends). Data are shown as mean ± SEM and p values of ≤ 0.05 were considered statistically significant.

### Suppression Assays

Cryopreserved Tregs, MulTregs and autologous Teff cells were thawed, labeled with 1μM DDAO (Treg/MulTreg) or Cell trace violet (CTV, Teff) and co-cultured in 96 well plates at the ratios indicated in the figure legends in the presence or absence of CD3/28 (1:50 bead to cell) for 5 days. Proliferation was measured by CTV dye-dilution within viable DDAO^-^CTV^+^ lymphocytes. Suppression was calculated relative to the proliferation in the absence of Treg or MulTreg cells. Alternatively, cryopreserved Tregs, MulTregs and allogeneic PBMCs were thawed, labeled with 10μM CPDe450 (Treg/MulTreg) or carboxyfluorescein succinimidyl ester (CFSE) (PBMC) and co-cultured in 96 well plates at the ratios indicated in the figure legends in the presence or absence of CD3/28 (Life Technologies) (1:10 bead to cell) for 5 days. Proliferation was measured by CFSE dye-dilution within viable CPDe450^-^CFSE^+^ lymphocytes. Suppression was calculated relative to the proliferation in the absence of Treg or MulTreg cells. In another experiment, CTV-labeled PBMC were stimulated with 100ng Influenza Hemagglutinin (Flu-HA, Revaxis) for 6 days in the presence or absence of autologous Treg or MulTreg cells and proliferation measured by dye-dilution within CD4^+^ CD3^+^ viable lymphocytes. Cytokine abundance in the supernatant of cell cultures was measured by the LEGENDplex™ multi-analyte flow assay kit, human Th cytokine mix & match subpanel (Biolegend).

### RNA-Seq Analysis

Six matched pairs of Treg and MulTreg were expanded and RNA extracted (RNeasy RNA extraction kit, Qiagen). Donors 2 and 5 correspond to matched pairs from T1D patients (**Figure 6B**). cDNA was then produced (SMART cDNA synthesis kit, Clontech) and DNA libraries produced using the Nextera XT kit (Illumina). Library quality was next determined by Bioanalyzer and sequencing performed by MiSeq using v3 chemistry and 150 cycle paired end reads. Sequencing reads generated from the Illumina platform were assessed for quality and trimmed for adapter sequences using TrimGalore! v0.4.2 (Babraham Bioinformatics), a wrapper script for FastQC and cutadapt. Reads were trimmed for quality using a Phred quality score cutoff of 20, implementing the algorithm used by BWA in which the cutoff score of 20 is subtracted from all qualities and the partial sum from all indices to the end of the sequence is calculated. The sequence read is then cut at the index at which the sum is at its minimum. After adapter removal and quality control trimming, reads with a minimum length of 20bp were then aligned to the human reference genome (GRCh37) using the STAR aligner v2.5.1 which was designed specifically for spliced alignment of reads from transcripts to a reference genome, using default parameters. The alignment for the sequences were guided using the GENCODE annotation for hg19. The aligned reads were analyzed for differential expression using Cufflinks v2.2.1, a RNASeq analysis package which reports the fragments per kilobase of exon per million fragments mapped (FPKM) for each gene. A total of 14,834 genes were identified to be expressed with any sample having FPKM >1. Differential analysis report was generated using Cuffdiff using the STAR alignments. By default, Cufflink normalizes the FPKM counts for each library by scaling the counts using the geometric means of the fragment counts across all libraries as described in Anders et al. (22). Differential genes were identified using a significance cutoff of q-value < 0.05 (FDR adjusted using Benjamini-Hochberg). For the HC, rows are centered; unit variance scaling is applied to rows. Rows are clustered using correlation distance and average linkage. The genes were then subjected to gene set enrichment analysis (GenePattern, Broad Institute) to determine any relevant processes that may be differentially overrepresented for the conditions tested. Additional custom gene set enrichment analysis and visualization were performed in R using the DOSE, enrichplot, and fgsea packages. STAR Aligner and Cufflinks were performed according to Dobin et al. and Trapnell et al. respectively (23, 24). For pathway analysis the Data (significantly impacted pathways, biological processes, molecular interactions, miRNAs, SNPs, etc.) were analyzed using Advaita Bio’s iPathwayGuide (https://www.advaitabio.com/ipathwayguide). This software analysis tool implements the ‘Impact Analysis’ approach that takes into consideration the direction and type of all signals on a pathway, the position, role and type of every gene, etc., as previously described (25–27). Bonferonni correction was used to identify pathways selected under highest stringency. Confirmatory pathway analysis of genes passing secondary Wilcoxon-paired tests was conducted with STRING functional protein association network analysis as indicated in the figure legend(s) (28).

### Xenogeneic GVHD Model

*Mice:* Immunodeficient NOD.Cg-Prkdcscid Il2rgtm1Wjl/SzJ (NSG) mice were purchased from Charles River Laboratories and maintained in a specific pathogen-free facility (Biological Services Unit, New Hunt’s House, King’s College London). All procedures were performed under sterile conditions in accordance with institutional guidelines and the Home Office Animals Scientific Procedures Act (1986) (Home Office license number: PPL 70/7302). *Xeno-graft vs host disease model:* 6-7-week-old NSG mice were injected intravenously with 1x10^7^ 3^rd^ party PBMCs ± Tregs in a 1:1 ratio. Control mice received saline alone. Following cell transfer, mice were monitored every 2-3 days for signs of xeno-GvHD. Parameters measured included weight loss, hunching, reduced mobility, ruffled hair, and orbital inflammation which were graded on a scale of 0-2 (29). Xeno-GvHD development/progression was scored in a blinded manner by two investigators and mice were sacrificed when pre-defined endpoints were reached including >15% weight loss and/or a summed clinical severity score of ≥7. Human CD45+ cell engraftment was measured by flow cytometry in peripheral blood obtained from tail bleeds every two weeks and the spleen following euthanasia. Mice were considered successfully engrafted and included in analyses when human CD45+ cells constituted >80% of total splenic lymphocytes.

### TSDR Analysis

Genomic DNA was isolated from d30 post-expansion Treg/MulTreg cells and d0 pre-expansion, sorted Teff cells using the Purelink™ Genomic DNA mini kit (Invitrogen, Life Technologies). Subsequently, 1μg of gDNA was sent to EpigenDx for bisulfate conversion and pyrosequencing to assess the methylation status of the *FOXP3* Treg- specific demethylated region (TSDR). The average methylation percentage of 9 CpG islands was calculated.

## Results

### MAPC Cell Co-Culture Results in Markedly Increased Treg Expansion

Based on the potential of clinical grade MAPC cells to promote Treg differentiation, we tested MAPC cell co-culture as a potential strategy to enhance Treg manufacture. To do so, we adopted the GMP compatible Treg process from the ONEstudy and ThRIL as a benchmark protocol (7, 9). CD4+ CD14- CD127lo CD25hi live Tregs were sorted from freshly isolated peripheral blood mononuclear cells (PBMC) of healthy volunteers (n=10) and stimulated 2:1 with anti-CD3/CD28 coated beads and maintained in the presence of 600IU/ml IL-2 and 125ng/ml rapamycin for 10 days, after which cells were re-plated with fresh anti-CD3/CD28 coated beads (**Figures 1A** and **S1A, B**). Following three sequential 10-day expansions, cells were starved of rapamycin and IL-2 for 48h, then harvested for endpoint analysis (cells expanded under these conditions are hereafter referred to simply as ‘Treg’). In parallel, we replicated these conditions with the addition of single donor, allogeneic MAPC cells at a ratio of 1:10 MAPC : Treg at day zero of each round. Treg cells generated under these conditions are hereafter referred to as ‘MulTreg’ (**Figure 1A**). At day 10, the number of MulTreg cells was 3.5 times greater than paired Tregs, 21.4 vs 6.1-fold expansion, respectively. This difference in yield increased to 9.6-fold by day 20 (440 vs 45.9-fold expansion) and further to 15.9-fold upon completion of the expansion at day 30 (11,178 vs 699.6-fold). The greater abundance of MulTreg cells was significant at all timepoints measured (q=3.9x10^-3^, Multiple Wilcoxon signed rank test) (**Figure 1B**). The yield at day 30 was greater (9/10 donors) or equivalent (1 donor) in MulTreg relative to Treg across the cohort (range 0.9-733-fold greater expansion in MulTreg vs Treg) (**Figure 1C**).

To validate these results, a separate research team at a commercial cell therapy facility in Leuven, (Belgium, EU) performed expansions in a second, independent validation cohort of 5 donors. Data produced at the Belgian research site confirmed that of the KCL team, demonstrating increased yield in 5/5 donors in MulTreg compared to Treg (range 2.3-7.7-fold, p=0.0313) (**Figure 1D**). This effect was found to be highly reproducible across 10/10 expansions run from a single donor (p=1x10^-3^ average 8.2-fold, range 1.7 to 20-fold) (**Figure S1C**), and when using four independent MAPC cell donors (**Figure S1D**). Combined analyses from both cohorts showed a significant, consistent, and marked increase in MulTreg yield relative to Treg at all timepoints (q=1.2x10^-4^), resulting in an average 9.4-fold increase in quantity by day 30 (**Figure 1E**). Indeed, MulTreg yields at day 20 were not statistically different from Treg yields at day 30 (p=0.094), suggesting equivalent dose could be reached 10 days earlier *via* the MulTreg platform. These data indicate that MAPC cell co-culture consistently results in a significantly more rapid expansion and greater yield of Treg cells during *in vitro* GMP-compatible platforms that emulate clinical manufacture protocols.

### MulTreg Exhibit Stable Treg Lineage Identity and a CCR7lo Integrin β7hi Phenotype

**Figure 2:**
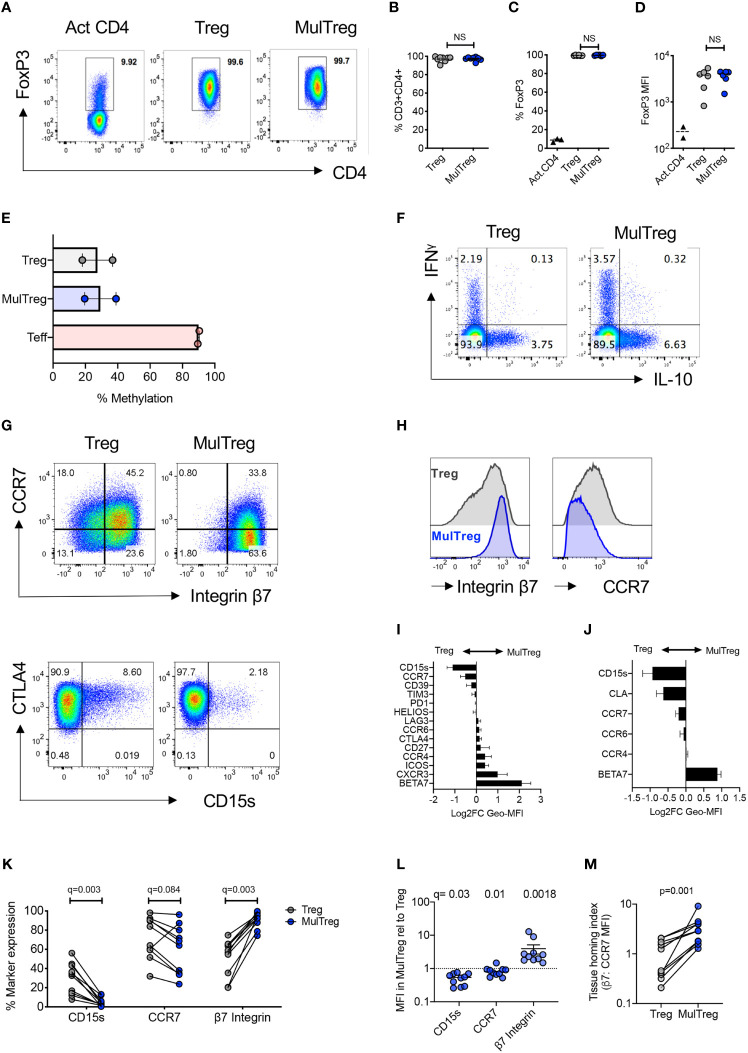
MulTreg are stably committed FoxP3hi CD4+ Treg cells exhibiting a distinct tissue homing phenotype. **(A)** Example of FoxP3 staining in activated CD4 T cells and Treg or MulTreg lines. **(B)** Frequency of CD4+ T cells in live gated events, n=8 pairs. **(C)** Frequency of CD4+ T cells expressing FoxP3 in activated bulk CD4 T cells (n = 2) Treg and MulTreg cell lines, n = 6 pairs. **(D)** Median fluorescence intensity of FoxP3 staining in activated CD4 T cells (n = 2), Treg and MulTreg cell lines, n = 6. **(E)** Mean percentage of methylation on 9 CpG islands of the *FOXP3* Treg-specific demethylated region (TSDR), n = 2. **(F)** Representative flow cytometry plots of expanded lines following restimulation for 5h with PMA/Io and intracellular staining with the cytokines indicated, gated on live CD4 T cells. **(G, H)** Representative flow cytometry plots **(G)** or histograms **(H)** of expanded lines showing expression of the markers indicated. **(I, J)** Relative MFI of markers indicated amongst Treg and MulTreg lines (expressed as log2FC in favor of MulTreg) from **(I)** n = 5 donors in the KCL (Discovery) cohort and **(J)** n = 5 donors in the Belgian (Validation) cohort. **(K–M)** Combined analysis of KCL and Belgian cohorts (n = 10) showing frequency **(K)** or relative MFI **(L)** of marker expression indicated and the ratio of β_7_ integrin to CCR7 MFI, defined as the tissue homing index **(M)**. Data points represent individual donors, Error bars = SEM of 5 or 10 **(L)** donors. Stats derived from single or multiple Wilcoxon matched-pairs signed rank test. NS, not significant.

The phenotype of cells harvested at day 30 was analysed by flow cytometry. Treg and MulTreg cells showed equivalent purity of CD3+ CD4+ T cells (mean Treg 96.9 ± 3.1% vs MulTreg 97.25 ± 2%, p=0.25, **Figures 2A, B**) that were >98% FoxP3 positive (mean Treg 99.35% ± 0.4, MulTreg 99.55% ± 0.2, p=0.44, **Figure 2C**) and expressed equivalent levels of FoxP3 Median Fluorescence Intensity (MFI) (mean Treg MFI 3366 ± 1654 vs MulTreg 3631 ± 1157, p=0.56), which was higher than T effector (Teff) cells, as anticipated (MFI 230.5 ± 87) (**Figure 2D**). These data were independently validated by the Leuven team in the validation cohort (**Figure S2A**, n=5). FoxP3 TSDR methylation analysis showed that, in contrast to Teff (mean methylation 89.9% ± 0.64) expanded Treg (27.4% ± 13.2) and MulTreg (29.25 ± 13.6) cells were stably committed to the Treg lineage (**Figure 2E**). Correspondingly, the frequency of effector (interferon γ (IFNγ), IL-17A, tumor necrosis factor α (TNFα), granzyme B and regulatory (IL-10) cytokines produced upon PMA/Io restimulation were low and comparable between Treg and MulTreg (p=ns for all, **Figures 2F** and **S2B**). These data demonstrate that MAPC cell-co-culture generates enhanced yields of highly pure and stably committed regulatory T cells.

The chemokine receptor profile of Tregs directs homing to inflamed tissues, whilst inhibitory receptor and differentiation markers co-define regulatory potential. We therefore assessed Treg differentiation, functional and homing markers in the KCL cohort. Of all markers tested the lymph node homing receptor CCR7 and CD15s (sialyl lewis x, expressed by Tregs in sarcoidosis) were lower in MulTreg, whilst gut homing integrin beta 7 (β_7_) showed a trend for increased expression in MulTreg according to both MFI and frequency (**Figures 2G–I** and **S2C**). Inhibitory receptors (e.g. CTLA-4, ICOS, PD-1), differentiation markers (e.g. Helios, CD27) and other chemokine receptors (e.g. CCR4) showed no difference in expression (**Figure S2C**). The Belgian research group independently validated and extended phenotypic analysis in the second cohort, illustrating the same trends for CD15s, CCR7 (both lower in MulTreg) and integrin β_7_ (higher in MulTreg) (**Figures 2J** and **S2D**). In addition, the skin homing marker CLA examined by the Belgian team was lower in MulTreg cells, suggesting that gut-specific, rather all tissue homing capacity was increased in MulTreg (**Figure S2D**). Combined analysis from the two cohorts (n=15) confirmed that the frequency (**Figure 2K**) and/or MFI (**Figure 2L**) of CD15s and CCR7 was significantly lower- whilst the gut homing integrin β_7_ was significantly and markedly higher in MulTreg cells. Finally, we derived a Treg tissue homing index from the ratio of MFI of integrin β_7_: CCR7 which was significantly higher in MulTreg compared to Treg (p=0.001, **Figure 2M**). These results demonstrate that MAPC cell co-culture generates pure, stably committed Tregs which maintain the expression profile of major inhibitory receptors and exhibit a distinct CD15slo CCR7slo β7hi phenotype.

### MulTreg Cells Suppress Human Antigen-Specific Th1- and Polyclonal Responses *In Vitro*

**Figure 3:**
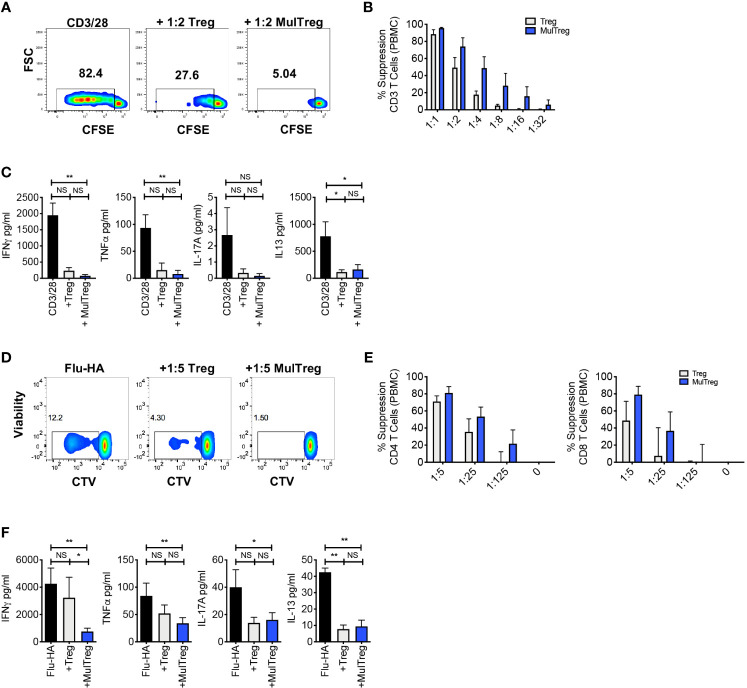
MAPC expanded Tregs exhibit superior suppression of antigen specific responses. **(A–C)** Suppression assay using 3rd party PBMC stimulated with CD3/CD28 microbeads for 6 days in the presence or absence of Treg or MulTreg lines. **(A)**. Flow cytometry plots showing proliferation in responder T cells from PBMC stimulated with CD3/CD28 microbeads (1:10 bead to cell) in the presence or absence of autologous, expanded Treg or MulTreg lines at a ratio of 1:2 Treg : PBMC. **(B)** Bar graph displaying % suppression of responder T cell proliferation at different ratios of Treg or MulTreg to PBMC. **(C)** Bar graph displaying cytokine levels in tissue culture supernatant from suppression assays in panel **(B)** (ratio 1:2 Treg : PBMC). n = 5. **(D–F)** Suppression assay using autologous PBMC stimulated with Flu-HA for 6 days in the presence or absence of Treg or MulTreg lines. **(D)** Flow cytometry plots showing proliferation in responder CD4 T cells from PBMC stimulated with Flu-HA in the presence or absence of autologous, expanded Treg or MulTreg lines at a ratio of 1:5 Treg : PBMC. **(E)** Bar graph displaying % suppression of responder CD4+ T cell proliferation at different ratios of Treg : PBMC, CD4 (left) and CD8 (right). **(F)** Bar graph displaying cytokine levels in tissue culture supernatant from suppression assays in panel **(E)** (ratio of 1:5). Error bars represent the SEM of 5 donors. Stats from Wilcoxon matched-pairs analysis **(B, E)** or Friedman test **(C, F)**. *p < 0.05, **p < 0.01, ns, not signficant.

Polyclonal *in vitro* suppression assays remain the gold standard release criteria for Treg cell therapy products, and the majority of immunopathology associated with autoimmunity and graft rejection is elicited by Th1/Th17 antigen-specific responses. We therefore evaluated the *in vitro* suppressive potential of Treg and MulTreg lines using a combination of polyclonal (anti-CD3/CD28 coated beads) and antigen specific, Th1 driven recall (Flu hemagglutinin vaccine ‘Flu-HA’) models. Both lines significantly suppressed T cell proliferation in polyclonally activated 3^rd^ party PBMC (n=5) (**Figures 3A–C**). During the polyclonal response, MulTreg, but not Treg co-culture led to significant reduction of IFNγ and TNFα whilst low levels of IL-17A secretion were not significantly reduced by either cell type (**Figure 3C**). In contrast, both lines inhibited IL-13 production (**Figure 3C**). The expansion of CD4 and CD8 T cells to Flu-HA was also attenuated by both Treg and MulTreg, with a trend for MulTreg to exhibit greater levels of suppression at 2 out of the 3 Teff: Treg ratios in CD4 and CD8 (**Figures 3D, E**). Whilst both cell products showed a clear trend towards inhibition of IFNγ, TNFα and IL-17A in Flu-HA responses this was only significant in MulTreg co-cultures, however, no difference was seen in inhibition of IL-13 (**Figure 3F**). In addition, MulTreg and Treg cells both suppressed responses to autologous, purified Teff CD4+ T cells (**Figure S3**). These data highlight that augmented expansion and phenotypic differences in MulTreg are accompanied by *in vitro* suppressive function that is equivalent to or greater than that observed with paired Treg.

### MulTreg Suppress Pathogenic Human Effector Responses During xGVHD *In Vivo*

**Figure 4:**
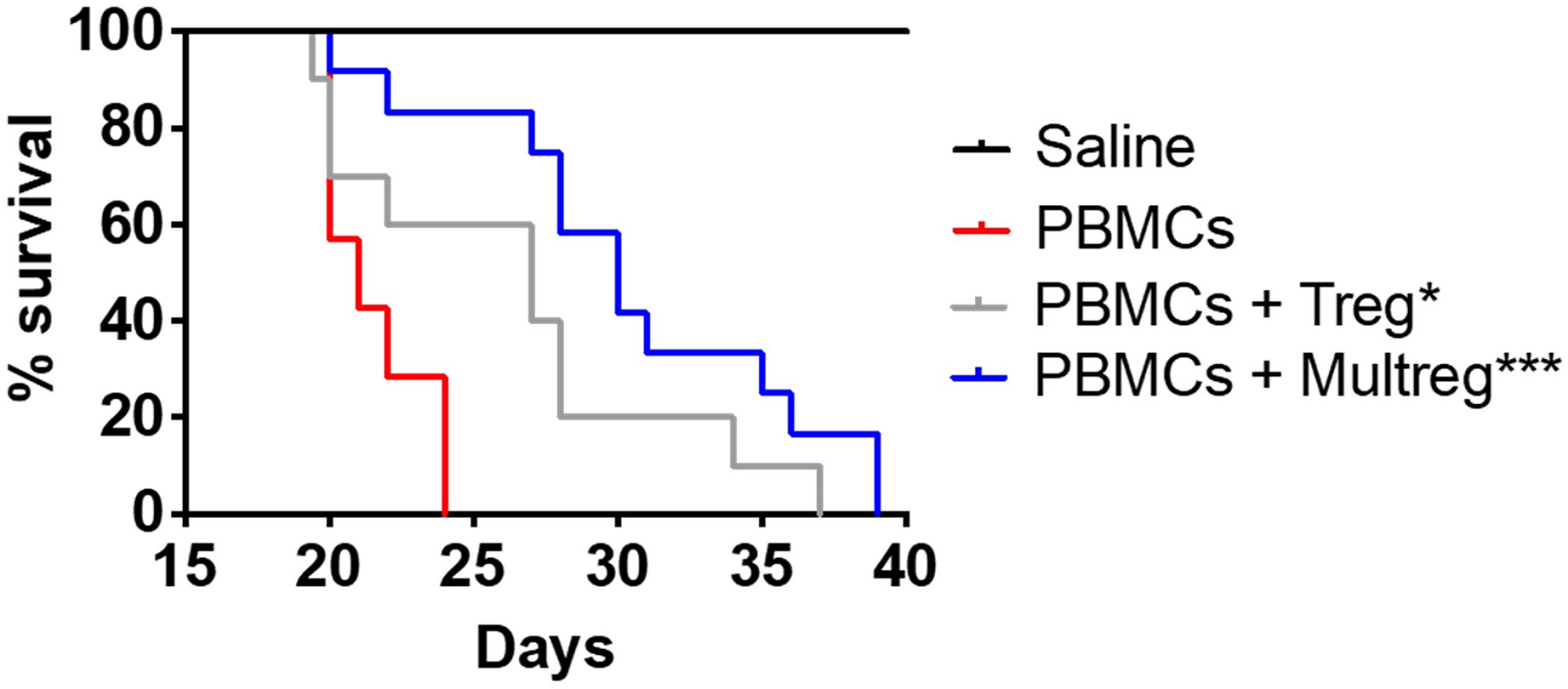
MulTreg effectively control the immune response in a humanized mouse model of xeno-GvHD. NSG mice were inoculated with 10^7^ PBMCs +/- 10^7^ Tregs or MulTregs or PBS as a control (PBMC alone n = 7, PBMC and Treg n = 10, PBMC and MulTreg n = 12, PBS alone n = 5). Survival of mice administered with MulTreg and Treg in addition to PBMC was significantly longer than those given PBMC alone. Log-rank Mantel-Cox Test (*p < 0.05, ***p < 0.001).

The *in vivo* regulatory capacity of MulTreg was assessed by their ability to suppress fatal immunopathology in a model of human into mouse xenogeneic GvHD (xGvHD). In this model, GvHD is driven by expansion of human T cells, which rapidly adopt an effector memory phenotype, a process that is dependent on xeno-reactivity with foreign MHC class-I and class-II molecules and resembles alloreactivity in the human transplant setting (30). The engrafted cells can be regulated by therapeutic manipulation making this a suitable model to test the suppressive capacity of Treg cell lines. In the absence of Treg administration, disease presented rapidly with no animals surviving beyond 24 days. Administration of Treg or MulTreg significantly slowed the progression of GvHD when compared to mice given PBMCs alone (median survival 27d Treg vs 21d PBMC; p=0.032 and 30d MulTreg vs 21d PBMC; p=0.003) with a trend for slightly higher survival with MulTreg compared to Treg (median survival 30d MulTreg vs 27d Treg; p=0.1) (**Figure 4**).

### MAPC Cell Co-Culture Facilitates Superior Expansion of Suppressive, Stable, β_7_hi Treg Cells From Patients With Autoimmune Disease

**Figure 5:**
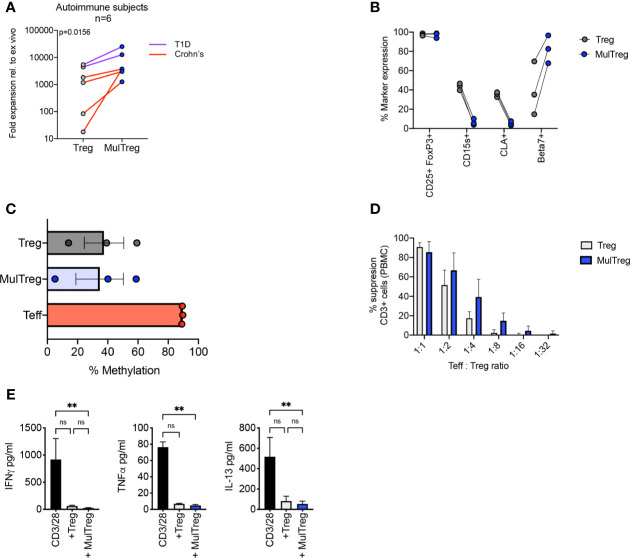
Characteristics of MulTregs from patients with Crohn’s Disease. **(A)** Average fold expansion (from *ex vivo*) for Treg and MulTreg lines grown from 4 individuals with Crohn’s disease and 2 individuals with type 1 diabetes (T1D). Wilcoxon matched-pairs signed rank test (n = 6). **(B)** Paired flow cytometric analysis of marker frequencies in Treg and MulTreg cells (n=3). **(C)** Average percentage of methylation on 9 CpG islands of the *FOXP3* Treg-specific demethylated region (TSDR) (n = 3). Friedman test (ns). **(D, E)** Suppression assay using 3rd party PBMC stimulated with CD3/CD28 microbeads (1:10 bead to cell) for 6 days in the presence or absence of Treg or MulTreg lines before analysis at d6. **(D)** Bar graph displaying % suppression of responder CD3+ T cell proliferation at different ratios of Treg : PBMC (n = 4). **(E)** Bar graph displaying cytokine levels in tissue culture supernatant from suppression assays in panel **(D)** (ratio 1:2) (n = 3), Friedman test. Data in **(A–D)** analysed *via* Wilcoxon matched-pairs analysis non-significant unless stated. **q < 0.01. ns, non-significant.

CD4+ CD25hi CD127lo cells were sorted from PBMC of patients with Crohn’s disease (CD) (n=4) and type-1 diabetes (T1D) (n=2) and expanded using the MulTreg protocol (patient demographic in **Table S1**). MulTreg from autoimmune patients expanded more rapidly than their Treg counterparts (mean 2159 vs 8239-fold relative to *ex vivo*, p=0.0156) (**Figure 5A**) and retained equivalent purity and expressed the characteristic CD15slo CLAlo β7hi phenotype (**Figure 5B**). As seen in healthy donors, Treg and MulTreg cells from CD patients harbored a demethylated TSDR (**Figure 5C**) and exerted suppression of polyclonal responses in PBMC according to proliferation (**Figure 5D**) and effector cytokine suppression (**Figure 5E**). These data suggest that MAPC co-culture can be used to rapidly expand a greater yield of suppressive, stably committed Treg cells from populations of patients with prototypic autoimmune disorders, from whom Treg expansion remains a challenge.

### MulTreg Proliferation and Phenotype Is Underpinned by Transcriptional Rewiring of Migratory and Replication Related Circuitry in Expanded Tregs

**Figure 6:**
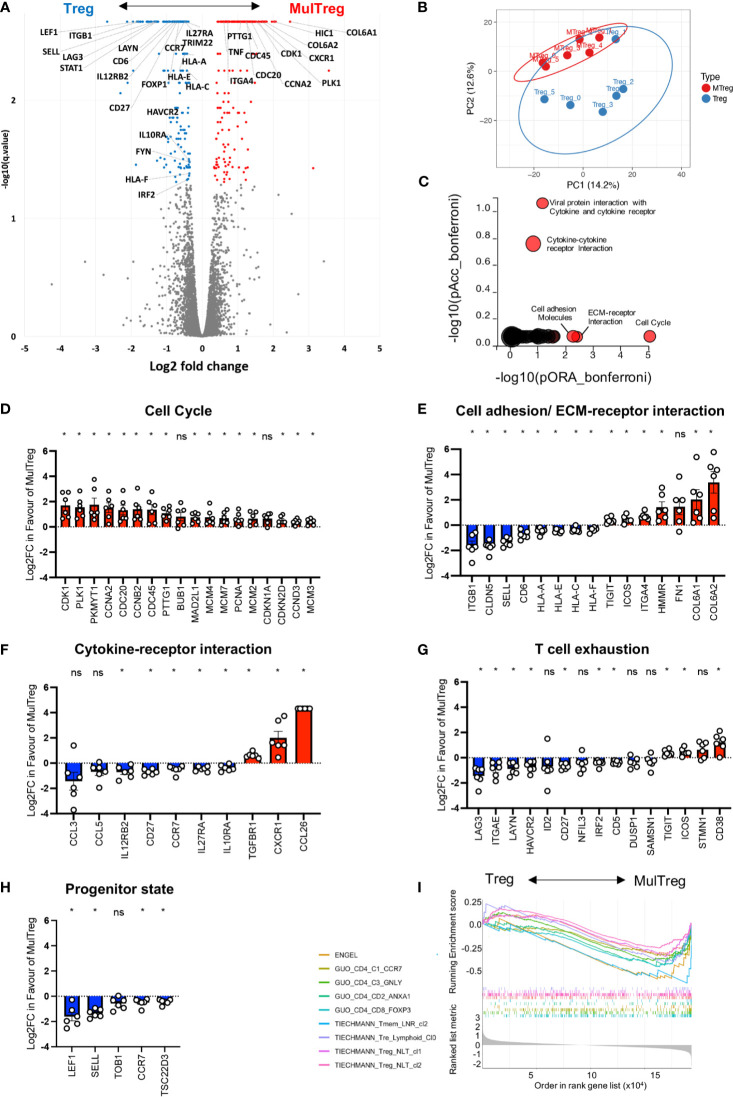
RNAseq analysis of Treg and MulTreg. Pairs of Treg and MulTreg expansions from n = 4 healthy donors and n = 2 patients with T1D were analysed by bulk RNAseq. **(A)** Volcano plot of all genes analysed highlighting differentially expressed genes (DEGs) (q < 0.05) in color, with genes of interest labelled. X axis represents log2 fold change in favor of Multreg. **(B)** Unsupervised principal component analysis showing global gene expression in all 14k transcripts. Plot shows the first two components (PC1 and 2) that account for 14.2% and 12.6% of the variance respectively. z PCs 3 and 4 accounted for 10.3 and 10%, respectively and PCs 5-11 contributed the remaining variance (range 9.2-6.5%). **(C)** IMPACT pathway analysis displaying pathways in which DEGs are significantly accumulated (pAcc) or over-represented (ORA) following Bonferroni correction. **(D–H)** Bar plots of genes in pathways highlighted by IMPACT analysis **(D–F)** or related to exhausted or progenitor T cell states **(G, H)**. **(I)** Gene set enrichment analysis (GSEA) Multiplot showing enrichment of the gene sets indicated from references in the main text. * < 0.05 Wilcoxon-paired test. Data points in **(D–H)** represent individual donors. ns, not signficant.

We next examined the transcriptional profile of the two cell types to understand the molecular mechanisms underpinning increased expansion and altered phenotype in MulTreg. The raw sequence data was uploaded to the Sequence Read Archive (SRA) database (Bioproject accession PRJNA751471). RNAseq analysis was performed on 6 additional paired Treg vs MulTreg expansions (n=4 healthy donor, n=2 patients with T1D). 363 genes were significantly differentially expressed (hereafter DEGs) after multiple correction testing (Benjamini Hochberg with an FDR of 0.05, Cuffdiff analysis -see *Methods*). Cell-cycle (*CDK1*, *CDC20*, *PLK1*) and migratory (e.g. *SELL, ITGB1, ITGA4, CXCR1*) loci were amongst those most differentially expressed, consistent with growth and phenotyping analysis, respectively, in addition to cytokine networks (*e.g. IL10RA, STAT1*, *IL12RB*), **Figure 6A**. Visualization of global gene expression in Principal Component Analysis (PCA) highlighted that MulTreg lines from multiple donors were transcriptionally more homogenous than paired Treg expansions (**Figure 6B**). Moreover, MulTreg exhibited a near uniform directional shift from Treg for each individual according to the first two principal components, suggesting a common pattern of molecular rewiring (**Figure 6B**). Pathway analysis revealed that MulTreg lines harboured a significant overrepresentation of transcripts involved in cell-cycle (19/95, q=0.002), **Figures 6C, D**, and modulation in an overlapping set of genes related to cell adhesion (11/53, q=0.031) and ECM-receptor interaction (6/14, q=0.0007), **Figures 6C, E**. In addition, we discovered a significant accumulation of perturbed genes associated with ‘viral protein interaction with cytokine and cytokine receptor’ (7/30, q=0.0006) and ‘cytokine-cytokine receptor interaction’ (11/77, q=0.002), **Figures 6C, F**, including *CCL26* which was highly expressed in MulTreg, but undetectable in Treg cells (**Figure 6F**). To ensure statistical robustness, we performed a subsequent round of Wilcoxon-Paired tests, validating 274/363 DEGs as significantly differentially expressed, including 39/44 (89%) of those within enriched pathways (Significance denoted in **Figures 6D–F**). Moreover, independent pathway analyses of the 274 validated DEGs confirmed that cell-cycle/DNA replication and ECM-receptor interaction pathways were enriched in MulTreg, whilst multiple cytokine signaling pathways connected to *STAT1* were diminished (**Figures S4A–C** and **S5A**).

Several DEGs were related to exhausted (*LAYN*, *LAG3*, *HAVCR2*, *ITGAE* lower in MulTreg, *ICOS*, *CD38*, *TIGIT*, higher in MulTreg; **Figure 6H**) and progenitor/quiescent (*LEF1*, *SELL*, *TOB1 CCR7*, *TSC22D3*; lower in MulTreg; **Figure 6H**) T cell differentiation programs. To further investigate this, we examined a total of 35 gene signatures from murine and human T cell subsets across several disease models/clinical indications by Gene Set Enrichment Analysis (GSEA). MulTreg exhibited a loss of quiescence, progenitor and lymph node homing gene modules (e.g. Elyahu et al. (31) resting Tregs in a pre-clinical ageing model; NES= -1.55, padj=0.0078, Engel et al. (32) TCF1+ progenitor Tregs that are precursors to gut homing progeny in a GVHD model; NES= -2.02, padj=0.0021, Miragaia et al. (33) memory lymph node resident memory T cells under homeostatic conditions; NES=-1.96, padj=0.0026) and human early differentiated and regulatory CD4 T cells in cancer, Guo et al. (34) (**Figure 6I**). Similarly, signatures of exhausted T cells were enriched in Treg vs MulTreg, including those from dysfunctional CD8 T cells in Melanoma (35) (NES= –1.71, padj=0.02) and, of relevance, functionally exhausted chimeric antigen receptor (CAR) Treg cells (36) (NES= -1.88, padj =0.0003), **Figure S5B**. Finally, we queried signatures of T cell subsets with disease related- or experimentally induced changes in Metabolism including Tregs from healthy donors vs autoimmune disease (37), or mice with T cell specific disruption of mTORC1 (38) or *Hif1a* (39). MulTreg were negatively enriched (NES=-1.68, padj=0.0015) for a signature of *Hif1a* deficient murine T cells, suggesting MulTreg may harness HIF1a associated glycolysis to a greater extent than counterpart Tregs, consistent with increased bioenergetic demand associated with elevated proliferation (39).

These data help to explain and extend our phenotypic and functional work, suggesting that MulTreg i) are transcriptionally poised for increased replication (*Hif1a* signature enrichment, diminished progenitor/quiescence or exhaustion signatures, cell-cycle pathway up-regulation, *CDK1*, *CDC20* expression), ii) exhibit a bias in homing properties that favor gut tissue localization (loss of *CCR7*, *SELL*, increased *CXCR1*, *ITGA4*), iii) are differentially sensitive to specific cytokine signals (loss of *IL27RA*, increased *TGFBR1*) iv) are enriched for certain co-stimulatory/inhibitory devices (*ICOS*, *TIGIT*), and v) produce distinct chemotactic molecules (e.g. *de novo* induction of *CCL26*).

## Discussion

The past decade has seen intense interest in evaluating the clinical utility of Tregs for a variety of indications including hematopoietic stem cell transplantation, autoimmune diseases, and solid organ transplantation (3, 37). While the results from these initial clinical trials have demonstrated the potential therapeutic value of Tregs, isolation and expansion of sufficient numbers of Tregs present major challenges due to reduced Treg frequencies and/or functionally defective Tregs reported in patients with autoimmune disorders (38–44). To overcome this deficiency, a variety of *ex vivo* strategies have been developed to select, isolate, and expand Tregs (4). Although progress has been made, there is still a significant need to develop more robust methods to manufacture clinical grade Tregs.

Numerous *in vitro* and *in vivo* studies have demonstrated that MAPC cells modulate uncontrolled immune responses and increase the proliferation of Tregs (17, 18, 21, 45, 46). Furthermore, a transient increase in Tregs was also observed in a clinical study evaluating the administration of MultiStem^®^, a clinical grade product of MAPC cells, in patients receiving a liver transplant (16). *In vitro*, MAPC cells promote Treg expansion *via* secretion of transforming growth factor β (TGFβ) and indoleamine 2, 3 dioxygenase (IDO) (47).

Herein, MAPC cells were introduced into a GMP compatible Treg expansion platform, resulting in greater expansion than paired Tregs but with similar Treg identity, stability, and function as demonstrated by FoxP3 expression, TSDR methylation status, and the ability to reduce both antigen specific autologous (Flu-HA), and polyclonal T cell proliferation (CD3/28) of autologous Teff or 3rd-party PBMC. The functional activity of MulTregs was further evaluated in a mouse model of acute xGVHD where it significantly delayed the xGVHD and extended survival of the animals compared to Tregs. Though differences between MulTregs and Tregs were non-significant in proliferation and xGVHD assays, we observed a trend towards greater potency of MulTreg both *in vitro* and *in vivo*. Why a greater difference in regulation was not seen between the two lines is unclear but may result from the benefits of proliferation and tissue homing being partly countered by reduced lymph node homing or decreased IL10RA and IL27RA expression in MulTreg. It is possible that the advantages of enhanced tissue homing would be maximized in human recipients where the full complement of chemotactic networks is intact. Moreover, as a better understanding of phasic and patient specific differences in Treg subset sensitivity are established, disease/stage dependent administration of tissue vs lymph node homing Tregs may be required, wherein MulTreg could offer a precision approach to rapidly target tissue damage once an excessive effector response has been mounted in the inflamed organ. To this end, it is known that for Treg therapy to be optimal, infused cells need to migrate to inflammatory sites where they can be activated in the target tissue (48). For example, intestinal tolerance requires gut homing and expansion of Tregs in the lamina propria (40). In this context, β_7_integrins guide intestinal T cell homing and retention (21). Indeed, whilst we have not formally replicated the results in the present study, we have previously demonstrated that β_7_ integrin positive Tregs preferentially migrate to human gut following adoptive transfer to a SCID mouse bearing subcutaneously implanted human small bowel (41). *ITGA4* is a marker of Beta 7 positive Tregs that facilitates formation of the alpha four beta 7 heterodimer, and CXCR1 is associated with Treg homing to the inflamed mucosa (42). Thus, together with loss of *CCR7*, *SELL* (lymph-node homing) and *CLA* (skin homing) the increased β_7_ integrin, *ITGA4* and *CXCR1* gene expression suggests a globally altered trafficking profile biased towards preferential gut homing, supporting potential clinical utility for the treatment of an autoimmune disorder of the gut. Therefore, we decided to evaluate the Tregs and MulTregs isolated from patients with CD; a debilitating autoimmune disorder of the gut that results in chronic inflammation. Treg treatment is being clinically evaluated in CD (48), however, isolation of Tregs from CD patients results in lower Treg numbers compared to healthy donors, which further supports the need to develop more robust expansion protocols for Tregs isolated from this patient population (43, 48). Comparable to the results with healthy Treg donors, MulTreg isolated and expanded from CD patients had greater expansion potential and higher levels of β_7_ integrin expression but similar FoxP3 methylation levels, and suppression of polyclonal stimulated T cell proliferation compared to Tregs, further underscoring their potential utility in CD.

Type 1 diabetes is another prototypic autoimmune disorder mediated by Th1 CD4 T cells and CTLs (34) where cell therapy approaches including MAPC cells (17, 18) and Tregs (47, 49, 50) show promise. The ability for MulTreg to suppress Th1-driven autologous, antigen-specific recall responses elicited by Flu-HA suggest that these cells can modulate key events in T1D immunopathology (48). Furthermore, like patients with CD, MulTreg cells appear more readily expandable from patients with T1D. Recent reports show that infused Treg cells decline from the circulation of patients with T1D at a highly variable rate, which has been suggested to result from trafficking, turnover, and/or exhaustion (26, 35). This implies that reduced exhaustion, well-defined homing profiles and/or increased cell banks for repeat dosing could be beneficial properties of an adoptive Treg therapy in T1D; highlighting a potential utility of MulTreg in T1D patients. Our data also suggest changes in cytokine receptor gene expression on MulTreg, which may include a shift from IL-10 and IL-27 induced regulatory function towards enhanced TGFBR1 signaling which has been shown to induce Treg retention in inflamed sites and control of experimental colitis (51). Moreover, loss of *IL12RB1*, which may account for reduced *STAT1*expression, could reduce the possibility of Th1 conversion in inflamed sites *in vivo* including the gut or pancreatic tissue in CD and TID, respectively.

CAR technology is currently being explored to enhance Treg specificity and functionality (5). Current CAR-Treg manufacturing approaches implement the initial steps of conventional polyclonal Treg expansion, rendering CAR-Treg manufacturing susceptible to similar manufacturing challenges (52). Given the promising results in polyclonal Treg expansion in the presence of MAPC cells, we believe that this approach could also be beneficial for CAR-Treg and antigen specific Treg manufacturing.

Our data suggests highly specific differences in functional molecules that could translate to an altered mechanism of suppression being harnessed by MulTreg. Firstly, we noted that the frequency of cells expressing PD-1, CTLA-4, CD39, LAG-3 and TIM-3 was not significantly different (FigS2C) consistent with MFI (Fig2I), although there were small but significant decreases observed in gene expression of LAG-3 and TIM-3 (*HAVCR2*), **Figure 6** e.g., ICOS was the only co-stimulatory marker found to be significantly higher in MulTreg by gene expression (**Figures 6E, G**) and MFI (**Figure 2I**). Since ICOS does not possess inhibitory potential *per se*, expression in MulTreg may reflect an increased sensitivity to stimulation *via* the ICOS pathway which could result in an altered secretome of regulatory molecules or the increased proliferation we observed, either directly *via* ICOSL or indirectly by representing an ‘effector-Treg’ (eTreg) phenotype more sensitive to IL-2 (53). Indeed, increased ICOS, enhanced proliferation, loss of CD62L, diminished *LEF1* and elevated CXCR3+ seen in MulTreg are characteristic of eTregs, which mediate immune control in peripheral tissues (54). Furthermore, *TIGIT* was also found to be significantly increased in MulTreg and is another feature of Th1 eTregs including those that infiltrate iselts in the NOD mouse model of T1D and upregulate CD5, like MulTreg (53). Together these data posit that increased TIGIT and ICOS may provide alternative mechanisms of stimulation and suppression of MulTreg, respectively, and combined with elevated CXCR3, loss of *SELL*, *LEF1* and enhanced proliferation are indicative of a Th1-eTreg-like phenotype. This is further underlined by the negative enrichment of *Hif1a* deficient T cells driven by enhanced glycolysis (29). Moreover, MulTreg display the gut homing phenotype of expanded Tregs previously shown to derive from adult PBMC, whilst imparting the proliferative phenotype seen in Tregs expanded from cord blood (48), reinforcing that MAPC-co-culture generates a Treg product with a novel spectrum of properties that resemble eTregs.

Like Tregs, MAPC cells also modulate immune responses by suppression of T cell activation and proliferation, secretion of anti-inflammatory factors such as TGFβ and IDO-1, and induction of M2 macrophages (18, 19, 47, 49). Further experimentation is warranted to evaluate the effect of MAPC cell and Treg adoptive co-transfer in a mouse model of acute xGVHD including assessment of the contribution of altered migratory profile and inhibitory/stimulatory receptors play in MulTreg mediated suppression *in vivo*.

In summary, the persisting challenges in adoptive T cell and Treg cell therapy include expansion failure from target patient populations, exhaustion, lack of purity and heterogeneity in cell product phenotype. MulTreg cells can potentially be developed as a therapeutic for autoimmune disorders including Crohn’s Disease, offering a means to rapidly expand a stable, less exhausted cell product with a defined, disease-relevant homing phenotype. Importantly, this can be achieved *via* addition of a clinical grade, off-the-shelf cell product to an existing GMP compatible process which has already demonstrated safety and shows preliminary efficacy.

## Data Availability Statement

The datasets presented in this study can be found in online repositories. The names of the repository/repositories and accession number(s) can be found below: NCBI [accession: PRJNA751471].

## Ethics Statement

Ethical review and approval was not required for the study on human participants in accordance with the local legislation and institutional requirements. The patients/participants provided their written informed consent to participate in this study. The animal study was reviewed and approved by King’s College London.

## Author Contributions

TT, AT, RD, and JR conceived and designed the project. TT, AT, GL, and JR supervised the project. JR, CH, VR, JB, EN-L, DB, and PB designed and executed experiments. All authors interpreted and analyzed data. JR, VR, CH, AV-T, AT, and TT wrote the manuscript. All authors contributed to the article and approved the submitted version.

## Funding

This research was funded/supported by the National Institute for Health Research (NIHR) Biomedical Research Centre based at Guy’s and St Thomas’ NHS Foundation Trust and King’s College London and/or the NIHR Clinical Research Facility. Work at King’s College London was supported by T1DUK Consortium Mechanistic Core funded by a grant from Diabetes UK (Ref: 15/0005232).

## Author Disclaimer

The views expressed are those of the author(s) and not necessarily those of the NHS, the NIHR or the Department of Health.

## Conflict of Interest

AT and AV-T are employees of Athersys Inc. and JB and VR are employees of ReGenesys BV a subsidiary of Athersys Inc. AT, AV-T, JB, and VR have compensated stock options from Athersys, Inc.

The remaining authors declare that the research was conducted in the absence of any commercial or financial relationships that could be construed as a potential conflict of interest.

## Publisher’s Note

All claims expressed in this article are solely those of the authors and do not necessarily represent those of their affiliated organizations, or those of the publisher, the editors and the reviewers. Any product that may be evaluated in this article, or claim that may be made by its manufacturer, is not guaranteed or endorsed by the publisher.

## Supplementary Material

Supplementary Figure 1 Details of Treg and MulTreg expansion. **(A)** Identification of Treg and Teff cells sorted analysis and expansion. **(B)** Example of isolated Treg cells from parent PBMC post sort, prior to expansion. **(C)** The fold expansion in biological replicates of Treg and MulTreg lines grown from 1 PBMC and 1 MAPC donor (n = 10). **(D)** The fold expansion in Treg and MulTreg lines grown from 1 PBMC donor using 4 different MAPC donors (p=0.065). Stats from Wilcoxon matched-paired signed rank test.

Supplementary Figure 2 Details of the expanded MulTreg lines. **(A)** Purity of Treg and MulTreg lines in the Belgian cohort. Frequency of CD4+ T cells expressing FoxP3 and CD25 (left), median fluorescence intensity of FoxP3 (centre), frequency of CD4+ T cells (right) in live gated events (n = 5). **(B)** Teff cytokine production in the KCL cohort, n = 5. **(C)** Paired analysis of marker frequencies in the KCL cohort (n = 10). **(D)** Paired analysis of marker frequencies in the Belgian cohort (n = 5). All stats from multiple correction adjusted Wilcoxon matched-pairs signed rank test, ns unless shown.

Supplementary Figure 3 Suppression assay using autologous PBMC stimulated with CD3/CD28 microbeads in the presence or absence of Treg or MulTreg lines. **(A)**. Flow cytometry plots showing proliferation in responder CD4+ T cells from PBMC stimulated with anti-CD3/CD28 for 6 days in the presence or absence of autologous, expanded Treg or MulTreg lines at a ratio of 1:25 Treg : PBMC. **(B)** Bar graph displaying % suppression of responder CD4+ T cell proliferation at different ratios of Treg : PBMC. **(C)** Bar graph displaying cytokine levels in tissue culture supernatant from suppression assays in panel B (at ratio of 1:5). Error bars represent the SEM of 6 donors. *p < 0.05, **p < 0.01.

Supplementary Figure 4 Validation of genes differentially expressed in Treg vs MulTreg. **(A)** Miniaturized heatmap representing 274 significant DEGs in Treg vs MulTreg determined after Wilcoxon tests. **(B)** STRING functional network analysis conducted on the inferred/putative protein set derived from the differential gene sets with DEG selectively expressed in MulTreg shown left and Treg, right. Interaction networks are shown for DEGs confirmed by Wilcoxon paired tests from transcriptional analysis of Multreg vs Treg lines (n = 6 pairs) from healthy donors (4 pairs) and patients with autoimmune T1D (2 pairs). Networks show proteins encoded by genes in each category (nodes) linked according to highest confidence functional and/or physical interactions (edges), with disconnected nodes removed. Known interaction (cyan from curated databases, magenta experimentally determined), predicted interactions (green = gene neighborhood, blue = gene co-occurrence), or textmining (lime), co-expression (black) or protein homology (grey) edges are shown. Node color is specific according to the functional enrichment of GO, Reactome or Kegg pathways shown in in the legend. Edge length is arbitrary. Network displayed in d) has been restricted to functional interactions only to make the plot legible. **(C)** Paired line plots for selected genes with significant Wilcoxon tests grouped manually according to annotations in STRING and IMPACT pathway analysis.

Supplementary Figure 5 **(A)** Annotated heatmap showing all 274 DEGs in Treg and MulTreg following Wilcoxon paired test validation. **(B, D)** Gene set enrichment analysis (GSEA) of the exhausted **(B)** and metabolic **(D)** gene sets indicated from references in the main text. **(C)** List of all GSEAs in the first round of GSEA analysis. Metabolic signatures were analysed in a separate round of analysis and are indicated in the main text.

Supplementary Table 1 Patient characteristics.

Supplementary Table 2 Ab panel Treg sort.

Supplementary Table 3 Ab panel Treg characterization.
