# Supplementary figures and images for: Augmented Expansion of Treg Cells From Healthy and Autoimmune Subjects via Adult Progenitor Cell Co-Culture
Source: Front Immunol. 2021 Sep 1;12:716606. doi: 10.3389/fimmu.2021.716606 (PMC8442662; doi:10.3389/fimmu.2021.716606)

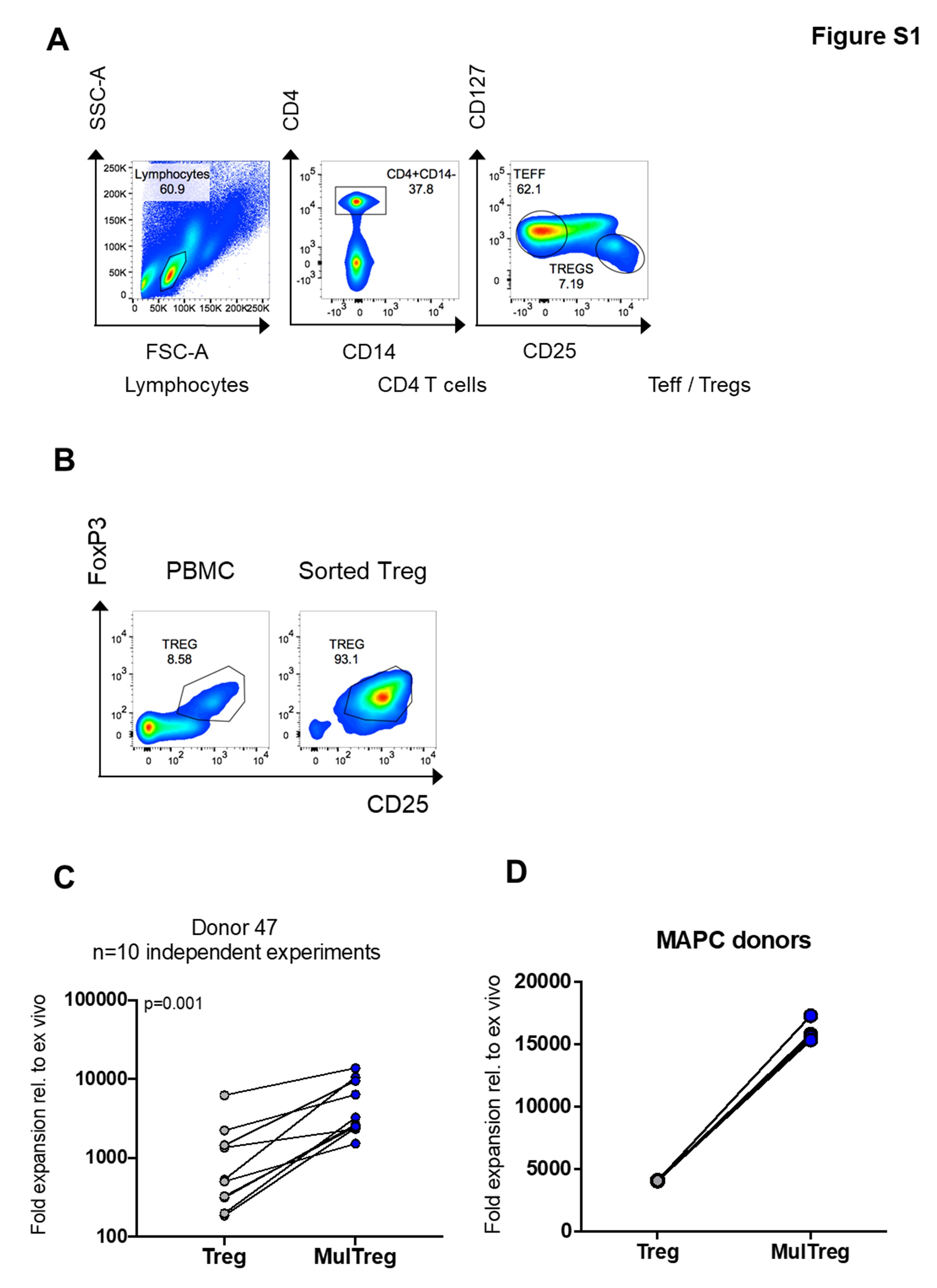

Supplement: Supplementary Figure 1 — Details of Treg and MulTreg expansion. (A) Identification of Treg and Teff cells sorted analysis and expansion. (B) Example of isolated Treg cells from parent PBMC post sort, prior to expansion. (C) The fold expansion in biological replicates of Treg and MulTreg lines grown from 1 PBMC and 1 MAPC donor (n = 10). (D) The fold expansion in Treg and MulTreg lines grown from 1 PBMC donor using 4 different MAPC donors (p=0.065). Stats from Wilcoxon matched-paired signed rank test. [file Image_1.jpeg]

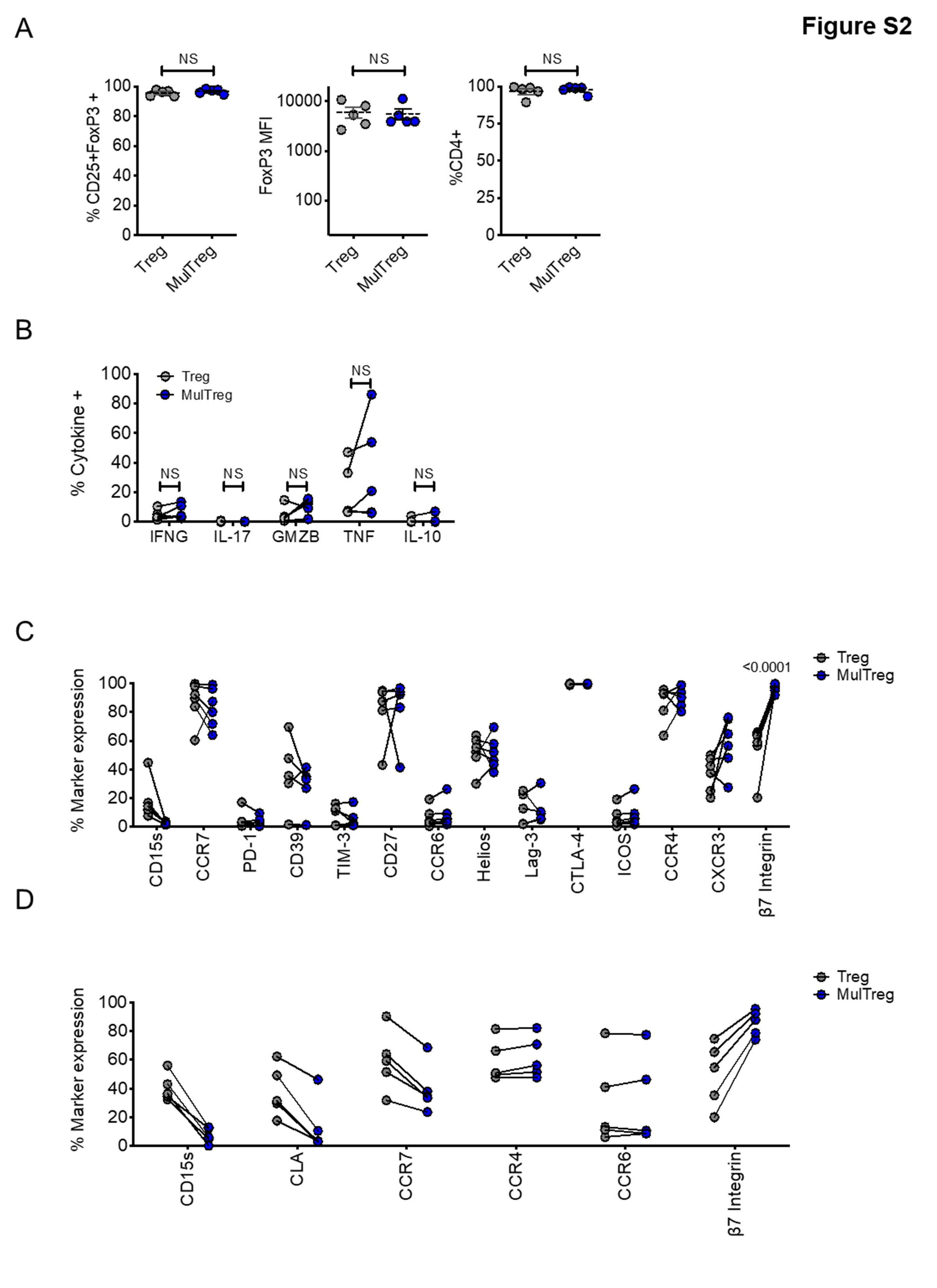

Supplement: Supplementary Figure 2 — Details of the expanded MulTreg lines. (A) Purity of Treg and MulTreg lines in the Belgian cohort. Frequency of CD4+ T cells expressing FoxP3 and CD25 (left), median fluorescence intensity of FoxP3 (centre), frequency of CD4+ T cells (right) in live gated events (n = 5). (B) Teff cytokine production in the KCL cohort, n = 5. (C) Paired analysis of marker frequencies in the KCL cohort (n = 10). (D) Paired analysis of marker frequencies in the Belgian cohort (n = 5). All stats from multiple correction adjusted Wilcoxon matched-pairs signed rank test, ns unless shown. [file Image_2.jpeg]

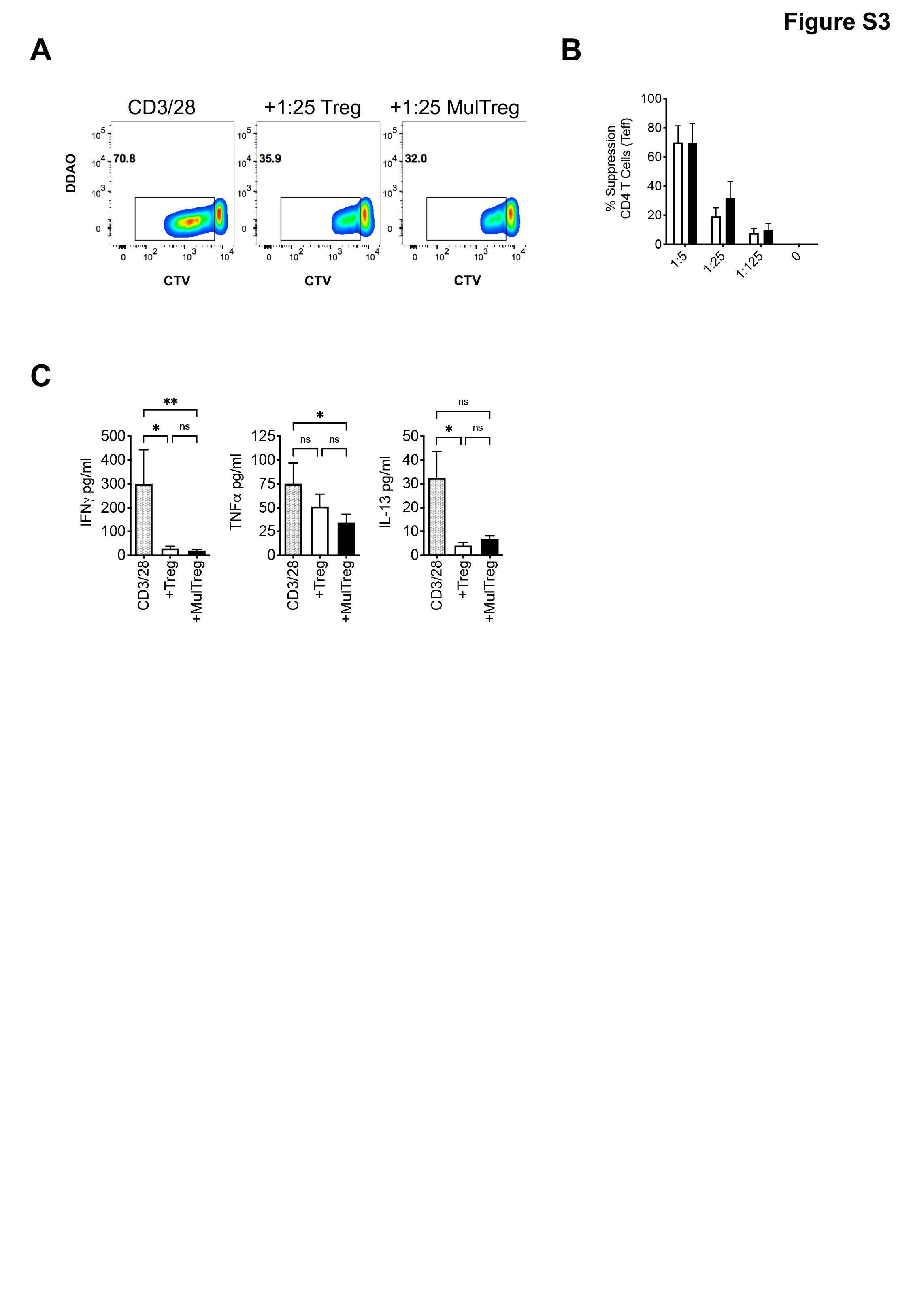

Supplement: Supplementary Figure 3 — Suppression assay using autologous PBMC stimulated with CD3/CD28 microbeads in the presence or absence of Treg or MulTreg lines. (A). Flow cytometry plots showing proliferation in responder CD4+ T cells from PBMC stimulated with anti-CD3/CD28 for 6 days in the presence or absence of autologous, expanded Treg or MulTreg lines at a ratio of 1:25 Treg : PBMC. (B) Bar graph displaying % suppression of responder CD4+ T cell proliferation at different ratios of Treg : PBMC. (C) Bar graph displaying cytokine levels in tissue culture supernatant from suppression assays in panel B (at ratio of 1:5). Error bars represent the SEM of 6 donors. *p < 0.05, **p < 0.01. [file Image_3.jpeg]

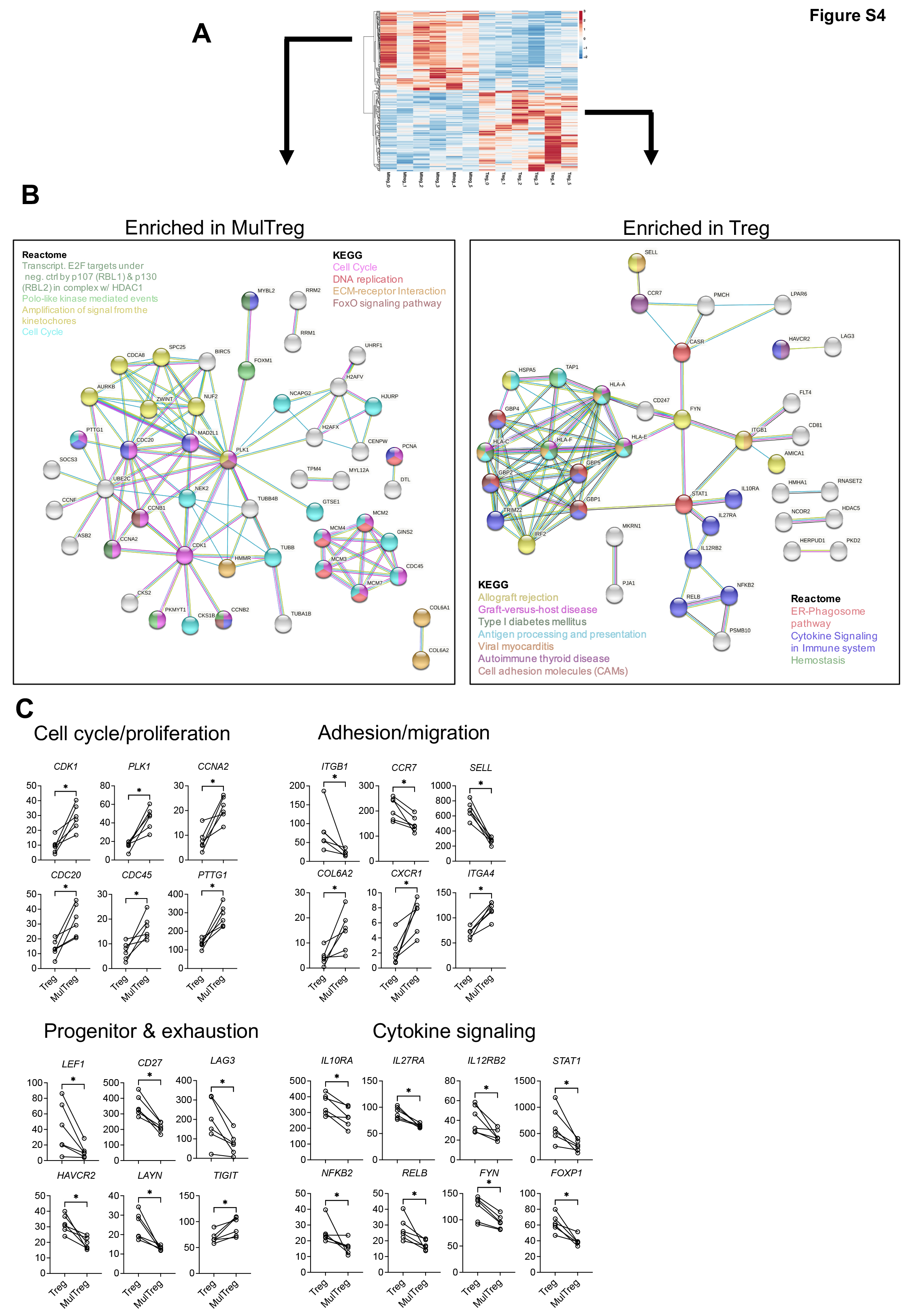

Supplement: Supplementary Figure 4 — Validation of genes differentially expressed in Treg vs MulTreg. (A) Miniaturized heatmap representing 274 significant DEGs in Treg vs MulTreg determined after Wilcoxon tests. (B) STRING functional network analysis conducted on the inferred/putative protein set derived from the differential gene sets with DEG selectively expressed in MulTreg shown left and Treg, right. Interaction networks are shown for DEGs confirmed by Wilcoxon paired tests from transcriptional analysis of Multreg vs Treg lines (n = 6 pairs) from healthy donors (4 pairs) and patients with autoimmune T1D (2 pairs). Networks show proteins encoded by genes in each category (nodes) linked according to highest confidence functional and/or physical interactions (edges), with disconnected nodes removed. Known interaction (cyan from curated databases, magenta experimentally determined), predicted interactions (green = gene neighborhood, blue = gene co-occurrence), or textmining (lime), co-expression (black) or protein homology (grey) edges are shown. Node color is specific according to the functional enrichment of GO, Reactome or Kegg pathways shown in in the legend. Edge length is arbitrary. Network displayed in d) has been restricted to functional interactions only to make the plot legible. (C) Paired line plots for selected genes with significant Wilcoxon tests grouped manually according to annotations in STRING and IMPACT pathway analysis. [file Image_4.jpeg]

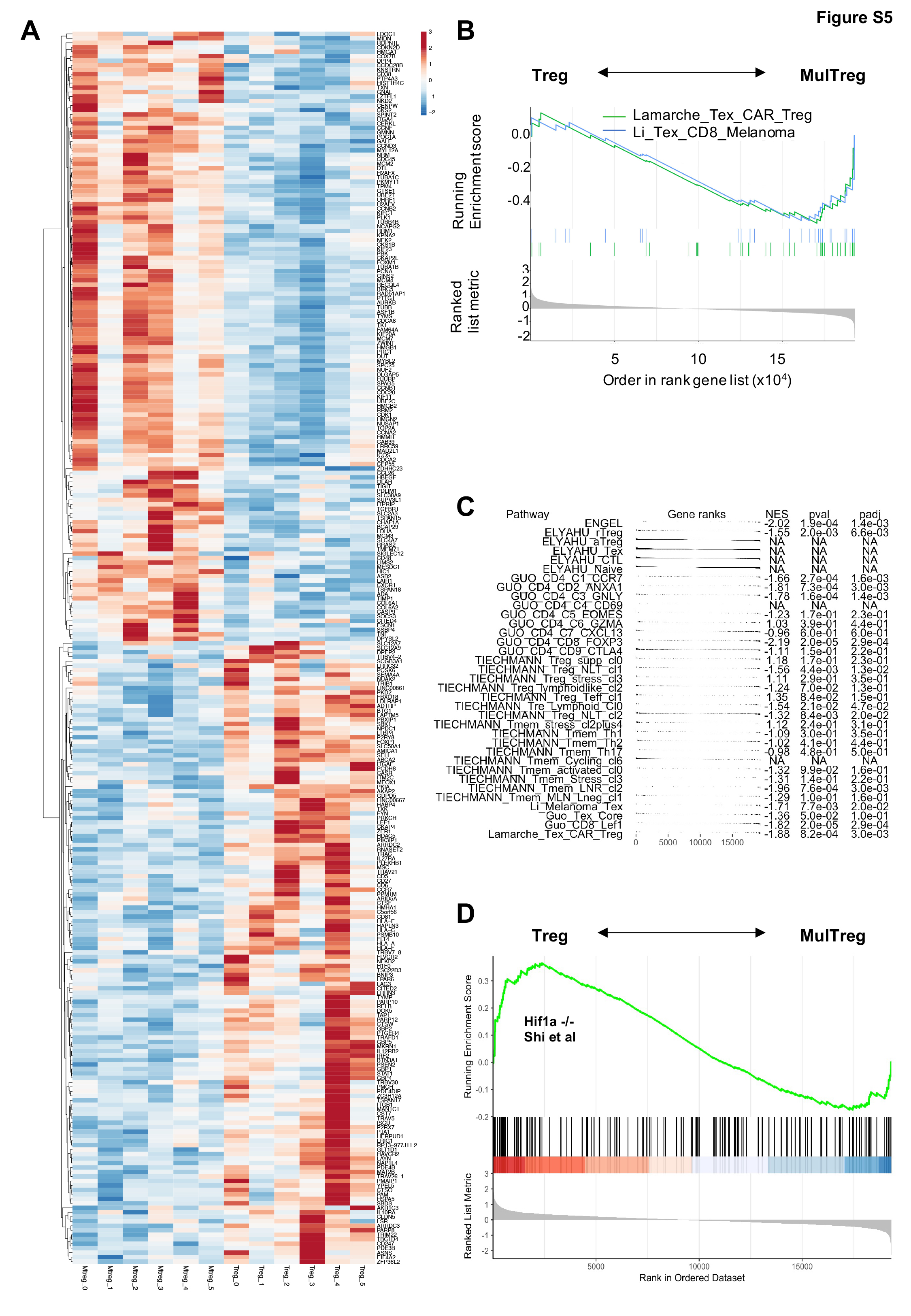

Supplement: Supplementary Figure 5 — (A) Annotated heatmap showing all 274 DEGs in Treg and MulTreg following Wilcoxon paired test validation. (B, D) Gene set enrichment analysis (GSEA) of the exhausted (B) and metabolic (D) gene sets indicated from references in the main text. (C) List of all GSEAs in the first round of GSEA analysis. Metabolic signatures were analysed in a separate round of analysis and are indicated in the main text. [file Image_5.jpeg]
